# Bars and medals

**DOI:** 10.7554/eLife.05787

**Published:** 2015-02-17

**Authors:** Eve Marder

**Affiliations:** Department of Biology and the Volen National Center for Complex Systems, Brandeis University, Waltham, United Statesmarder@brandeis.edu

**Keywords:** Living science, careers in science, scientific publishing, scientific excellence

## Abstract

There can only be one gold medal in each event at the Olympics. In science, on the other hand, as **Eve Marder** explains, it is more important to recognize excellence in its many different forms than it is to identify a winner.

In high jumping and pole vaulting, the phrase ‘raising the bar’ means exactly that: the bar is raised higher and higher until there is just one athlete left in the competition. The height of the bar is, therefore, an objective measure of performance and a reasonable way to determine the winner of the competition. However, I have been struck by how often I hear the phrase ‘raising the bar’ when discussing, for example, students taking exams or faculty being considered for tenure and promotion.

While it is tempting to use the metaphor of the ‘bar’ as a way of asking whether a person is ‘good enough’ to merit promoting (or if a manuscript is ‘good enough’ to merit publishing), this facile comparison hides many essential differences between deciding who wins the gold medal in the Olympic pole vault competition and what is important when judging excellence in science. In the pole vault, there can only be one winner in each competition. Moreover, the criterion for naming the winner is unitary, agreed upon, and entirely unambiguous. Nothing we do in science is analogous. We are, we hope, promoting many great scientists and publishing many great papers. The purpose of a tenure and promotion committee or an editorial board is not to find the single gold medalist, but to recognize excellence, often across many dimensions, including some that are difficult or impossible to quantify.

Of course, there are instances in academic science in which there can be only one successful candidate. If a department is doing a search for a new assistant professor, for example, there is often only one position available at a given time. However, it is not uncommon for the first choice candidate to decline the position. More importantly, if search committees are honest with themselves, they will usually acknowledge that there are multiple exceptional candidates who differ in many qualities, and that the decision is rarely so simple as ‘Who is better?’. More often the relevant question is: ‘Who is the best fit for what we are trying to achieve, since we are forced to make a choice?’

Some journal editors and reviewers use the metaphor of either the 100 meter sprint or the Marathon, and ask nothing more than, ‘Is this author the first to cross the finish line with a new finding?’ But again, unlike the Olympics, it is usually not clear where the finish line is, or who decides where it is. Of course, there are rare cases where the finish line is obvious, as in who was first to purify the nicotinic acetylcholine receptor, or to solve the crystal structure of an ion channel. But we don't fill journal pages with papers about such clearly defined big successes. And in the long run, being first may not be as important as getting it right. More importantly, when two labs are working simultaneously on an important problem, and arrive at similar conclusions within a short time of each other, the ‘second’ paper actually strengthens the conclusions of the first, so it is important that the second paper is published just as quickly as the first and that the authors receive their fair share of recognition. In today's world, rife with concerns about ‘failures to replicate’, we should celebrate the replication of an important finding, not penalize those authors for coming in second.

In the long-run being first may not be as important as getting it right.

Bars are also rigid barriers. Bars on windows and doors can imprison us or exclude us from entering places we might otherwise wish to explore. As we think about ‘raising the bar’ as a metaphor for standards for academic performance, we should not forget that each time we do so, we create barriers of one kind of another. Sadly, some among us take comfort that they are special because they are part of a privileged and restricted community, not because they have accomplished exceptional work. It is often they who talk about ‘bars’ because they are protecting turf or trying to convince themselves of their own worth.

In our zeal to reward excellence by awarding prizes and selecting individuals for the gold medals of science, we should be careful to remember that all findings in science build on work of less-honored people, and that all medals and prizes should properly celebrate the reality that, as a community, we have made significant progress in understanding our world. For every gold medal awarded in science to highly deserving individuals, there are many equally deserving individuals who have not been so honored. Medals and prizes enhance our individual and collective credibility in the broader community, just as the Olympics increase the public interest in many relatively obscure sports like luge or curling. But we should always value the athletes who compete for the joy of their sport, and the many thousands of our colleagues who work in science to participate in the joy of discovery who will never be medalists but who, nonetheless, are heroes.

At eLife we are privileged that we can publish all of the outstanding papers that we have the wisdom to recognize, and we eschew the notion of ‘the bar’ as we search for excellence. To the extent to which our editors are both visionary and rigorous, we will succeed in publishing many papers that change the way we understand the world (Malhotra and Marder, 2015). To the extent to which departments and universities are both visionary and rigorous, they will succeed in appointing and retaining people of diverse skills, interested in exploring a wide variety of problems. The dangers of linear assessments of accomplishments, as done in the pole vault, are that we run the risk of narrowing our sense of what achievement in science means and, as a result, narrowing the kinds of experiments that are done to those that can be evaluated by an overly simple (if easily understood) metric.

**Figure 1:**
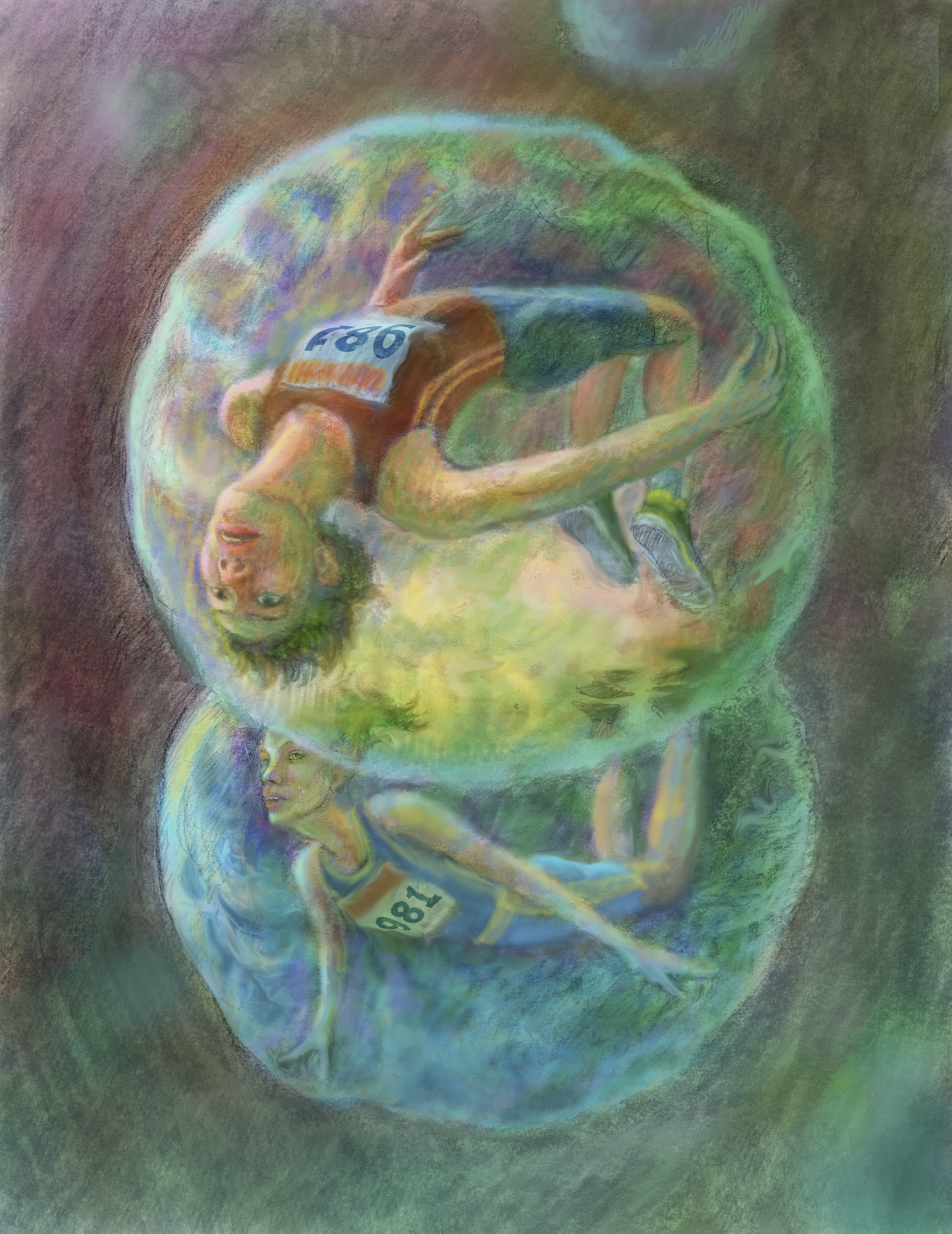
The notion of ‘the bar’ is not useful in science.

ILLUSTRATION: BEN MARDER
